# Systematic Approach for the Formulation and Optimization of Solid Lipid Nanoparticles of Efavirenz by High Pressure Homogenization Using Design of Experiments for Brain Targeting and Enhanced Bioavailability

**DOI:** 10.1155/2017/5984014

**Published:** 2017-01-23

**Authors:** Shweta Gupta, Rajesh Kesarla, Narendra Chotai, Ambikanandan Misra, Abdelwahab Omri

**Affiliations:** ^1^Department of Pharmaceutical Technology, Parul University, Vadodara, Gujarat, India; ^2^Department of Pharmaceutics, Oxbridge College of Pharmacy, Bangalore, India; ^3^Department of Pharmaceutics, A. R. College of Pharmacy, Vallabh Vidyanagar, Anand, Gujarat, India; ^4^Pharmacy Department, Faculty of Technology and Engineering, M. S. University of Baroda, Gujarat, India; ^5^Department of Chemistry & Biochemistry, Laurentian University, Greater Sudbury, ON, Canada

## Abstract

The nonnucleoside reverse transcriptase inhibitors, used for the treatment of HIV infections, are reported to have low bioavailability pertaining to high first-pass metabolism, high protein binding, and enzymatic metabolism. They also show low permeability across blood brain barrier. The CNS is reported to be the most important HIV reservoir site. In the present study, solid lipid nanoparticles of efavirenz were prepared with the objective of providing increased permeability and protection of drug due to biocompatible lipidic content and nanoscale size and thus developing formulation having potential for enhanced bioavailability and brain targeting. Solid lipid nanoparticles were prepared by high pressure homogenization technique using a systematic approach of design of experiments (DoE) and evaluated for particle size, polydispersity index, zeta potential, and entrapment efficiency. Particles of average size 108.5 nm having PDI of 0.172 with 64.9% entrapment efficiency were produced. Zeta potential was found to be −21.2 mV and the formulation was found stable. The* in-vivo* pharmacokinetic studies revealed increased concentration of the drug in brain, as desired, when administered through intranasal route indicating its potential for an attempt towards complete eradication of HIV and cure of HIV-infected patients.

## 1. Introduction

According to the World Health Organization, approximately 35 million people worldwide are living with HIV/AIDS including 3.2 million children of less than 15 years age. And an estimated 2.1 million individuals worldwide are newly infected with HIV every year [1]. Since the beginning of the epidemic, almost 78 million people have been infected with the HIV virus and about 39 million people have died of HIV. AIDS is the sixth leading cause of death among people aged 25–44 in the United States [2]. Current therapies with antiretroviral drugs are effective in reducing plasma viral levels but are ineffective in eradicating the virus from other sites like CNS due to their inability to reach and accumulate in certain cellular and anatomical reservoirs where virus potentially harbors. The CNS is the most important HIV reservoir site [3]. Due to the restricted entry of anti-HIV drugs, the brain is thought to form a viral sanctuary site. This not only results in virological resistance, but also is often associated with the development of complications such as progressive deterioration in mental function, symptoms of motor abnormalities, mild neurocognitive disorder (MDR), HIV associated dementia (HAD), HIV encephalitis (HIVE), and even death in many cases [3–6].

Efavirenz is a nonnucleoside reverse transcriptase inhibitor (NNRTI) of choice and is recommended as a first-line antiretroviral drug used in the high activity antiretroviral therapy (HAART) for the infections of human immunodeficiency virus [7]. Efavirenz (EFV) is a highly lipophilic drug of BCS class II having water solubility of 9.2 *μ*g/mL (pH 8.7) at 25°C and 4.6 as the log *p* value [8, 9]. Because of low water solubility of the drug, extensive first-pass metabolism, and metabolism by enzymes, low bioavailability (40–45%) of the drug has been reported [9–11].

Solid lipid nanoparticles (SLN) are gaining increased attention during recent years because of various advantages over other colloidal drug delivery systems like increased drug stability, increased protection of drug against enzymatic metabolism, possibility of controlled drug release, high drug loading capacity, biocompatibility, ease of large-scale production and sterilization, less variability in release mechanisms and their kinetics, potential for increased permeability due to lipid and surfactant contents and, hence, enhanced bioavailability, and ligand-mediated or passive targeting due to their small size through oral, parenteral, dermal, nasal, ocular, and pulmonary routes of administration [12–14].

Improved permeability of the drug is observed due to solubilization of endothelial cell membrane lipids and membrane fluidization because of surfactant effect [15]. There are tremendous possibilities in the anti-HIV drug delivery using SLN as carrier [16, 17]. SLN of various drugs are being investigated for brain targeting [18, 19]. When administered intranasally, the small nanoparticles penetrate through the mucosal membrane by paracellular or transcellular route. The transcellular process is responsible for the transport of lipophilic drugs that show a rate dependency on their lipophilicity [20]. The close connection between the olfactory bulb and the cerebrospinal fluid (CSF) offers a potential route for nasally delivered drugs to the CSF, provided that the drug is able to cross the nasal epithelium and the arachnoid membrane. Trigeminal pathways deliver a low molecular weight drug from the nose to the brain [21]. Both small and large molecules can pass rapidly from the nose into the brain along olfactory nerves and trigeminal nerve structures, without primarily passing via the CSF [22].

In the present investigation, an attempt was made to design and formulate solid lipid nanoparticles of the antiviral drug, efavirenz, to increase their bioavailability and overcome the challenges associated with the drug like low oral bioavailability due to extensive first-pass metabolism, low solubility, high protein binding, metabolizing enzymes, and efflux mechanisms [23, 24]. The nanoparticles of efavirenz were also proposed to target the drug to brain and increase its bioavailability in brain when administered through intranasal route.

The noninvasive intranasal administration route was proposed to offer rapid onset of action, no first-pass effect, no gastrointestinal degradation which in turn has the potential to improve the bioavailability by transport of drug through olfactory route and integrated nerve pathways bypassing the blood-brain barrier and allowing the direct transport of drug from nose to the brain.

## 2. Materials and Methods

### 2.1. Materials

Efavirenz and Tenofovir disoproxil fumarate were obtained as gift sample from M/S Sun Pharma Ltd., Sikkim, India, and from Paradise Healthcare, Vadodara, India, respectively. Lipids like glyceryl tripalmitate (tripalmitin), glyceryl monostearate, glyceryl tristearate, and surfactants—poloxamers (Pluronic F68 and Pluronic F127)—were purchased from Sigma Aldrich. Compritol 888 ATO (glyceryl behenate) was obtained from Gattefosse, France. Solvents like acetonitrile and methanol were purchased from Merck and ethyl acetate from Spectrochem Pvt. Ltd., Mumbai, India.

### 2.2. Methods

#### 2.2.1. Design of Experiments

Product should be designed to meet patients' needs and the intended product performance.

Pharmaceutical development should include defining of quality target product profile (QTPP), identifying and determining potential critical quality attributes (CQAs), selecting an appropriate manufacturing process, defining a control strategy, and identifying through, for example, prior knowledge, experimentation, and risk assessment, the material attributes, and process parameters that can have an effect on product CQAs. The systematic approach could facilitate product development and continual improvement and innovation throughout the product lifecycle [24, 25]. Investigations were done by factorial design to minimize particle size and maximize the encapsulation efficiency of the drug in SLN.

#### 2.2.2. Selection of Lipid

Different lipids were screened on the basis of solubility studies. The solubility of the drug was determined in different lipids. Amount of drug dissolved in known amount of each lipid at a temperature 5°C above the melting point of the respective lipid was determined using digital shaker water bath (NOVA Instruments Pvt. Ltd., Ahmedabad, India) and the lipid showing maximum solubility for the drug was proposed to have maximum drug loading capacity and was selected for further investigations [19].

#### 2.2.3. Selection of Surfactant

With the selected lipid, nanoparticles were prepared using different surfactants and were evaluated with respect to the particle size, PDI, and entrapment efficiency. The particle size and PDI were determined using Malvern Zetasizer Nanoseries Nano-ZS, UK. Selection of the surfactant was made based on minimum particle size and PDI with maximum entrapment efficiency.

#### 2.2.4. Drug-Excipient Compatibility Study

IR spectra of pure drug and the physical mixtures of drug and selected excipients stored at 25 ± 2°C, 60% ± 5% relative humidity for a period of 7 days were recorded using FT-IR spectrophotometer (Bruker Alpha-One, Bruker Optik, Germany) in the range of 4000–500 cm^−1^ and compared for any significant change [26, 27].

#### 2.2.5. Selection of Formulation Technique

Various techniques for SLN formulation are high shear homogenization and ultrasound, high pressure homogenization, solvent emulsification and evaporation technique, microemulsion based SLN preparation technique, and so forth [12, 28–30]. The selection of the technique was made based on the evaluation of particle size, PDI, and entrapment efficiency of the nanoparticles obtained with the trial batches using the commonly used and reported to be reliable and powerful techniques.

*(1) High Pressure Homogenization*. There are two general homogenization techniques (hot homogenization and cold homogenization) which can be used for the production of SLN [12]. In the present study, hot homogenization technique was investigated. The drug was incorporated into the melted lipid. The drug loaded lipidic phase was dispersed in a hot aqueous surfactant solution under continuous stirring to form a coarse o/w emulsion. It was then homogenized at the temperature above the melting point of the lipid using high pressure homogenizer (Panda Plus/GEA Niro Soavi, Parma, Italy) to form o/w nanoemulsion which was cooled to room temperature for solidification and formation of solid lipid nanoparticles [31, 32]. 

*(2) Solvent Evaporation Method*. The lipophilic drug was dissolved in a water-immiscible organic solvent and was emulsified in an aqueous phase containing the surfactant under continuous stirring on a magnetic stirrer. The organic solvent was evaporated and nanoparticulate dispersion was formed by precipitation of the lipid in the aqueous medium [12].

#### 2.2.6. Optimization of Process Variables

On the basis of literature survey and a few trial batches, various critical process variables which may have significant effect on the critical quality attributes were identified for each step involved in the formulation and were subjected to optimization. Preliminary optimization of stirring time, RPM, and temperature was done by conducting the experiments at three levels of each process variables involved during stirring of the hot aqueous surfactant solution while adding the drug incorporated lipidic phase for the formation of coarse emulsion. Critical process variables involved during the high pressure homogenization were optimized using 3^2^ factorial design with Design Expert 9.0.3.1 software (Stat-Ease, Inc., USA). The pressure and number of cycles were selected as independent variables and the response on particle size and PDI were investigated. Sonication time and amplitude were optimized for the sonication of the nanoparticulate dispersion after homogenization.

#### 2.2.7. Optimization of Formulation Variables

3^2^ factorial design was employed for optimization of formulation variables and Design Expert 9.0.3.1 software was used for statistical analysis by ANOVA, generating model equations and constructing contour plots and 3D surface plots for each response. Amount of drug with respect to lipid and concentration of surfactant were investigated as independent variables at three levels and the critical quality attributes selected were particle size, PDI, and entrapment efficiency as responses.

#### 2.2.8. Evaluation of Optimized Formulation

*(1) Particle Size, Polydispersity Index (PDI), and Zeta Potential*. The average particle size, PDI, and zeta potential of the solid lipid nanoparticles were determined using Zetasizer Nanoseries Nano-ZS, Malvern Instruments, Malvern, UK. Dynamic light scattering, DLS, and Laser Doppler Electrophoresis were used for the determinations of particle size and for zeta potential. The samples were put in “folded capillary cells” and results obtained for size, PDI, and zeta potential were recorded.

*(2) Entrapment Efficiency*. Entrapment efficiency was determined by determining the amount of free drug spectrophotometrically at 247 nm in the supernatant after centrifugation of the known amount of nanoparticulate dispersion at 10000 RPM using REMI centrifuge (BL-135 R) for 15 minutes. The entrapment efficiency was calculated using the equation [33]

(1) Entrapment efficiencyAmount of entrapped drugAmount of total drug×100=Weight of drug added in the formulation−Weight of free drugWeight of drug added in the formulation×100=WT−WFWT×100.

*(3) Transmission Electron Microscopic Evaluation*. The surface morphology of the optimized SLN was investigated using transmission electron microscope. Briefly, it was carried out by operating at an acceleration voltage of 200 kV. A drop of SLN dispersion was placed on grid. Approximately 2 min after sample deposition (1-2 *μ*L), the grid was tapped with filter paper to remove surface water and air dried. The image was taken using transmission electron microscope with CCD camera (TEM Philips Tecnai 20, Holland). 

*(4) Histopathological Studies*. Histological studies were carried out using isolated goat nasal mucosa. Freshly isolated goat nasal mucosa was sectioned into three pieces. One piece was treated with PBS pH 6.4 (as negative control), the other with a mucociliary toxicity agent—isopropyl alcohol (as positive control)—and the third one with the SLN dispersion [34]. After 24 hours, all the samples were washed properly with distilled water, fixed, processed for dehydration, embedded into paraffin wax, and stained with hematoxylin and eosin. DPX was used as mounting medium and microtoming was performed using microtome (model 0126, Yorco, India). The histopathological examinations for determination of damage/irritation due to the formulation were performed using inverted microscope (Nikon TS-100) [35, 36]. 

*(5) Drug Release Profile*.* In-vitro* drug diffusion profile was obtained by dialysis-bag/dialysis-sac method [34]. SLN dispersion and plain drug suspension were filled in activated dialysis membrane bags (dialysis membrane 110 (LA 395), HiMedia, cutoff 12000 Da) and suspended in glass beakers containing methanolic phosphate buffer saline (PBS) (pH 6.4, 50% v/v). Efavirenz has limited solubility in buffer but is soluble in methanol; hence methanol was added to PBS pH 6.4 to maintain the perfect sink conditions [34, 37]. The beakers were placed on magnetic stirrers and stirred with magnetic beads and were covered with paraffin film to prevent any evaporative loss during the experimental run [38]. Aliquots were withdrawn from the receptor compartments at periodic time intervals for 24 hours and replaced with equivalent amounts of fresh diffusion medium. The aliquots were analyzed spectrophotometrically at 247 nm. All the experiments were performed in triplicate.

#### 2.2.9. *In-Vivo* Studies

*In-vivo* studies were performed on adult Wistar albino rats. A protocol for animal studies was approved by Institutional Animal Ethics Committee (IAEC) and Committee for the Purpose of Control and Supervision of Experiments on Animals (CPCSEA) (protocol number PIPH 04/15 CPCSEA921/PO/Ere/S/05/CPCSEA). Animals were housed in polypropylene rat cages. Rice husk was used as the bedding material. Laboratory rat pellet feed and pure drinking water were supplied ad libitum. The rats were divided into two groups. Group I (test group) consisting of 6 animals were administered with a total volume of 0.25 mL of the developed SLN formulation (equivalent to 0.06 mg efavirenz), divided into five small volumes of 0.05 mL each administered within a 5-minute interval intranasally [34, 39]. The second group (standard) consisting of 6 animals were given the marketed formulation—EFAVIR—efavirenz capsules IP orally (powder equivalent to 25 mg efavirenz from capsule dispersed in 1 mL water).

The plasma samples from each animal were collected and the animals were sacrificed by an overdose of pentobarbital sodium at 24 hours. The brains were isolated, weighed, homogenized in PBS pH 6.4 at 5000 rpm using Silent Crusher M homogenizer (Heidolph, Germany), and centrifuged and the supernatants were collected for determination of drug concentration [15]. The amount of drug in plasma and the brain homogenate were determined by the method developed and validated for estimation of efavirenz in plasma using HPLC (unpublished work). The lower limit of quantification for the HPLC method in detecting the drug in plasma was 0.05 *μ*g/mL. Tenofovir disoproxil fumarate was used as internal standard. Brain : Plasma ratio, bioavailable fraction, and relative bioavailability were calculated using the formula

(2) Brain : plasma=Conc. of drug in brainConc. of drug in plasma ,Bioavailable fraction=Bioavailable doseAdministered dose ,Relative bioavailability=Systemic availability of drugsystemic availability of an oral standard of same drug.

#### 2.2.10. Stability Studies

The stability of the formulation was assessed under different storage conditions as per ICH guidelines, namely, 5 ± 3 and 25 ± 2°C/60 ± 5% RH [40–42]. The samples were evaluated at 0, 0.5, 1, 2, 3, and 6 months for physical appearance, average particle size, PDI, and zeta potential. All the studies were conducted in triplicate.

#### 2.2.11. Data Analysis

The data obtained were analyzed statistically using *t*-test and ANOVA. The data obtained for the optimization of process and formulation variables were statistically analyzed by analysis of variance (ANOVA) with in-built software design of Design Expert 9.0.3.1 software (Stat-Ease, Inc., USA). It was used to determine the significance and the magnitude of the effects of different variables and their interactions. Probability values less than 0.0500 were considered as statistically significant.

## 3. Results and Discussions

### 3.1. Design of Experiments

Literature survey was done to identify and determine the QTPP, CQAs, manufacturing procedures for SLN, various processes, and formulation attributes having effect on product CQAs. For the intended solid lipid nanoparticulate drug delivery system, almost all routes of administration—oral, parenteral, dermal, ocular, nasal, and so forth—have been reported for topical, systemic, or central nervous system actions. For better absorption of the drug, minimum average size of the nanoparticles is desired. Hence, minimum particle size is one of the most important CQAs along with minimum PDI (monodispersity), maximum entrapment efficiency, minimum zeta potential of ±20 mV for stability [26], and no residual solvent for avoiding toxicity and ensuring safety. A CQA is a physical, chemical, biological, or microbiological property or characteristic that should be within an appropriate limit, range, or distribution to ensure the desired product quality [43, 44]. In the present investigation, different preliminary experiments were performed for selection of suitable excipients/materials which may directly and/or indirectly influence critical quality attributes. The compatibility of the drug-excipient was checked before the optimization of various process and formulation variables. With the selected excipients, various critical process variables identified through the literature were optimized sequentially as per the steps involved in the formulation. Finally, the formulation variables were optimized using 3^2^ factorial design and Design Expert software for analyzing the data statistically and graphically using response surface plots [44].

### 3.2. Selection of Lipid

**Figure 1:**
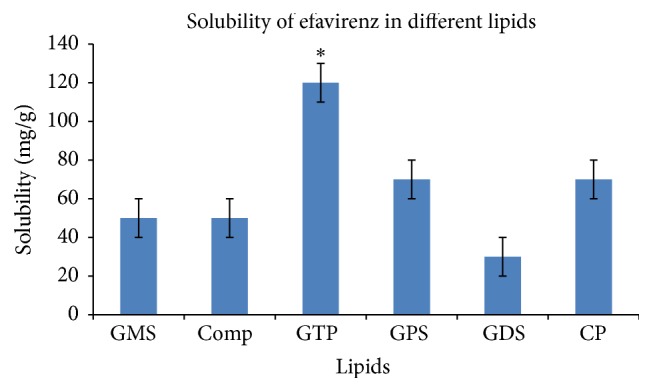
Solubility of efavirenz in different lipids [GMS: glyceryl monostearate, Comp: Compritol 888 ATO (glyceryl behenate), GTP: glyceryl tripalmitate (tripalmitin), GPS: glyceryl palmitostearate, GDS: glyceryl distearate, CP: cetyl palmitate].

Improved permeability of the drug is reported due to lipid content in SLN [11]. SLN contain lipids which remain solid at room temperature and body temperature. The lipids are pure triglycerides (tristearin, tripalmitin, trimyristin, etc.), long-chain alcohols (cetyl alcohol), waxes (bees wax, cetyl palmitate), and sterols (cholesterol) [45]. The selection of the lipid was primarily based on the solubility of the drug in lipid since the higher the solvent capacity is, the higher the drug loading potential will be [46]. The solubility of the drug was determined in six different lipids— glyceryl monostearate, glyceryl behenate (Compritol 888 ATO), glyceryl tripalmitate (tripalmitin), glyceryl palmitostearate, glyceryl distearate, and cetyl palmitate. The results obtained are as shown in Figure 1.

Glyceryl tripalmitate (tripalmitin) showed maximum drug solubilizing capacity of 120 ± 10 mg drug/gram of lipid while glyceryl palmitostearate and cetyl palmitate showed the next highest solubilizing capacity of 70 ± 10 mg drug/gram of lipid. Thus, the solubility of drug in glyceryl tripalmitate was found to be significantly higher than other lipids (*p* < 0.05). Medium and long-chain fatty acids are also reported to have good solvent capacity in comparison to short-chain fatty acids. Moreover, triglycerides have many advantages as the foundation of lipid-based delivery systems. They are commonly ingested in food, fully digested and absorbed, and therefore do not present any safety issues [46]. Tripalmitin also has GRAS status as per 21 CFR § 186.1555 and hence was selected for further investigations. Glyceryl tripalmitate is also reported to be used for SLN preparation during various investigations [18, 47, 48].

### 3.3. Selection of Surfactant

SLN are the colloidal system of nanoparticles made up of solid lipids as matrix medium which is stabilized in aqueous media by surfactants [15]. Solubilization of endothelial cell membrane lipids and membrane fluidization due to surfactant effect lead to improved permeability [46]. For the selection of surfactant, nanoparticles were prepared with six different surfactants using tripalmitin as lipid and were evaluated for particle size, PDI, and entrapment efficiency. The results obtained are as shown in Table 1.

Poloxamer 188 (Pluronic F68) was found to give minimum particle size and PDI with maximum entrapment efficiency. The difference in particle size was found to be statistically significant in comparison to others (*p* < 0.05) while the differences observed in PDI and entrapment efficiency were statistically nonsignificant. Poloxamers are nonionic triblock copolymers composed of a central hydrophobic chain of polyoxypropylene (poly(propylene oxide)) flanked by two hydrophilic chains of polyoxyethylene (poly(ethylene oxide)). Poloxamers are also reported to sterically stabilize the nanoparticles and reduce the adsorption plasma proteins or opsonins on the surface of nanoparticles by providing hydrophilic property to the surface of nanoparticles and thus can prevent the clearance of drug containing SLN from circulation [15]. In general, bulky and nonionic surfactants are reported to be less toxic than single-chain and ionic surfactants [45]. Hence, poloxamer 188, also having GRAS status, was selected for further investigations for the formulation.

### 3.4. Drug-Excipient Compatibility Study

**Figure 2:**
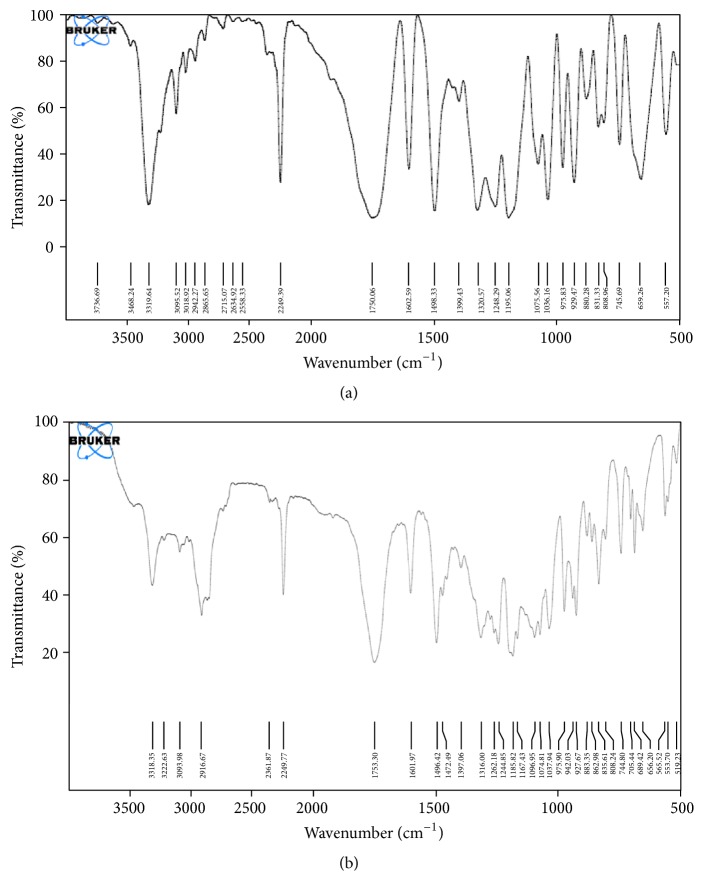
IR spectra of drug and physical mixture of drug and excipients. (a) IR spectrum of drug (Efavirenz). (b) IR spectrum of drug (efavirenz) + lipid (tripalmitin) + surfactant (poloxamer 188).

IR spectra of pure drug efavirenz and the physical mixtures of efavirenz, tripalmitin, and poloxamer 188 are shown in Figure 2.

It was observed from Figure 2(a) that all major peaks of the drug were obtained in the IR spectra as shown in Table 2 and no significant change was observed in the IR spectra of drug-excipients mixture (Figure 2(b)) indicating the compatibility of the drug with the selected excipients [7].

### 3.5. Selection of Formulation Technique

SLN formulations were prepared by different techniques (as trials) and were compared with respect to particle size, PDI, and entrapment efficiency. The results obtained are as shown in Table 3.

It was observed that lower particle size with higher entrapment efficiency was obtained by high pressure homogenization. The differences in particle size and entrapment efficiency obtained with two techniques were found statistically significant (*p* < 0.01) while the difference in PDI was statistically insignificant. The results were found to be in accordance with the findings reported in the literature [12, 28]. There are no chances of residual organic solvent since the high pressure homogenization technique avoids the use of organic solvent. The use of organic solvents presents a major toxicological disadvantage with solvent evaporation technique [28, 49]. Another advantage of the high pressure homogenization technique is its scale-up feasibility as it easily allows a laboratory, pilot, or large-scale production [19]. Hence, further investigations were carried out with high pressure homogenization technique.

### 3.6. Optimization of Process Variables

Various critical process variables which may have significant effect on the critical quality attributes were identified for each step involved in the formulation, that is, stirring, high pressure homogenization, and sonication. Preliminary optimization of stirring time and RPM of high speed homogenizer (Heidolph Silent Crusher) were carried out by conducting the experiments at three different RPM (5,000 to 10,000) for three time durations (10 to 20 minutes) at room temperature. From the results obtained (results not shown) in terms of particle size and PDI, it was observed that as the homogenization time and/or homogenization speed increased, the particle size decreased which may be attributed to increased force of deforming droplets at higher speed leading to smaller particles [50]. The observation was in accordance with reported results [34]. It was also observed that with the increase in homogenization time PDI also increased which may be due to formation of foam and more aggregation in the formulation. Best results were obtained by stirring at 10,000 RPM for 15 minutes. Hence further investigations were done with homogenization/stirring speed of 10000 RPM for 15 min.

To study the effect of temperature on the performance attributes, particle size and PDI were determined for the investigations carried out at three different temperatures (60–80°C). Best results were obtained at 70°C. It may be because the lipid would have remained at melted condition at this temperature and the temperature is much below the melting point of the drug as well; hence drug degradation due to temperature remains negligible. In general, higher temperatures result in lower particle sizes due to the decreased viscosity of the inner phase [51].

Critical process variables involved during the high pressure homogenization were pressure and number of cycles. These were optimized using 3^2^ factorial design with Design Expert 9.0.3.1 software (Stat-Ease, Inc., USA). Thirteen runs were carried out with pressure (500 to 900 bars) and number of cycles (3 to 7) as independent variables at three levels and particle size and PDI as dependent variables. Full factorial design used for optimization of process variables is shown in Table 4.

ANOVA was applied to determine the significance and the magnitude of the effects of the variables and their interactions. ANOVA for Response Surface Quadratic Model for Response 1 (particle size) and Response 2 (PDI) are shown in Tables 5(a) and 5(b), respectively. As observed from Table 5(a), for Response 1 (PS), the model *F* value of 42.26 implies that the model is significant. There is only a 0.01% chance that a “model *F* value” could occur due to noise. Values of “Prob > *F*” less than 0.0500 indicated that model terms are significant. In this case, *A* (pressure), *B* (number of cycles), and *A*^2^ are significant model terms. Values greater than 0.1000 indicate that the model terms *AB* and *B*^2^ are not significant.

Full model equation for particle size in terms of coded factors was obtained as

(3) PS=+389.62−91.18∗A−40.95∗B+11.73∗AB−31.16∗A2−5.16∗B2.

Final reduced polynomial equation for particle size in terms of coded factors was obtained as

(4) $$\begin{array}{c} \text{PS}=+388.14-91.18*A-40.95*B-33.13*A^{2}. \end{array}$$

As shown in Table 5(b), ANOVA results confirmed the adequacy of the quadratic model (model Prob > *F* is less than 0.05) for PDI. The individual effect of number of cycles (*B*) was found to be significant (*p* value < 0.05) and of pressure (*A*) was marginally significant (*p* value = 0.0536).

Full model equation for PDI in terms of coded factors was found to be

(5) PDI=+0.31−0.042∗A−0.063∗B−2.000E−003∗AB−5.914E−003∗A2+0.071∗B2.

Final reduced polynomial equation for PDI in terms of coded factors

(6) $$\begin{array}{c} PDI=+0.31-0.042*A-0.063*B+0.069*B^{2}. \end{array}$$

**Figure 3:**
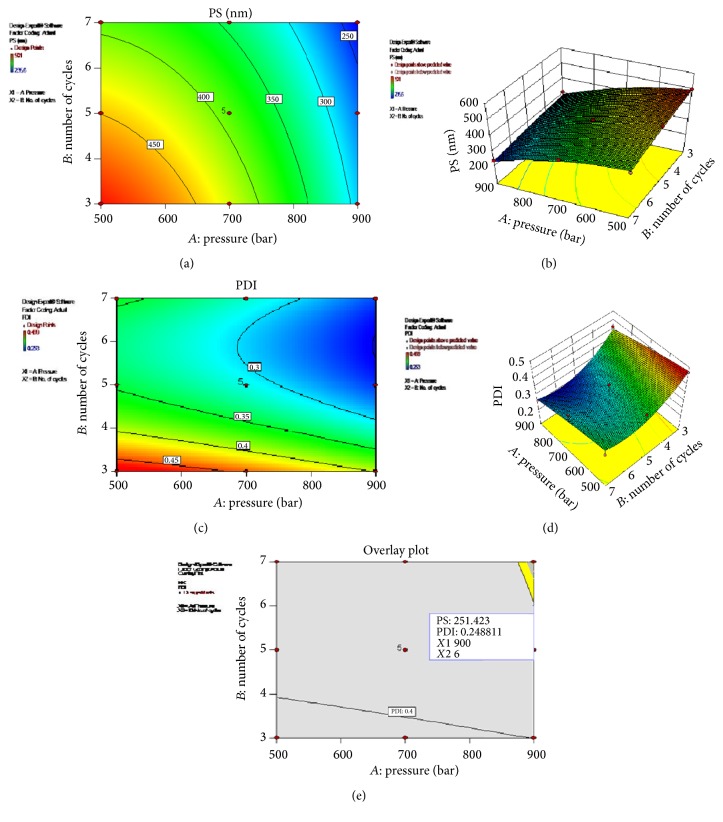
Contour plots, 3D surface plots, and overlay plot for process variables. (a) Contour plot for particle size. (b) 3D surface plot of particle size. (c) Contour plot for PDI. (d) 3D surface plot of PDI. (e) Overlay plot for optimization.

The regression model obtained was used to generate the contour plots, 3D surface plots, and the overlay plot for particle size and as shown in Figure 3 for analyzing interactions of the independent factors. It was observed from Figures 3(b) and 3(d) that as the pressure and/or number of cycles were increased, the particle size and PDI were found to reduce. The results were found in contrast to the investigations for production of SLN with cetyl palmitate as the solid lipid where particle size and PDI was observed to be increased with increase in pressure [14].

#### 3.6.1. Experimental Validation of Design Space (for Process Variables during High Pressure Homogenization)

The multidimensional combination and interaction of input variables and process parameters that have been demonstrated to provide assurance of quality are termed as the design space [52]. Design space could be determined from the common region of successful operating ranges for the two responses and is depicted with the yellow region in the overlay plot (Figure 3(e)). Experimental validation of DoE trials was undertaken by preparation and characterization of nanoparticles at the check point batch suggested by software. The observed values (particle size 259.7 nm and PDI 0.220) were in close agreement with the predicted values (particle size 267.274 nm and PDI 0.263276) and established the reliability of the optimization procedure. For further reduction in particle size, the effect of sonication was also investigated and sonication time and amplitude were optimized. The experiments were carried out at three amplitudes for three time durations (data not shown). No significant effect was observed with change in amplitude. Particle size was found to reduce with increase in sonication time, but PDI was increased with increased time. Homogenization followed by ultrasonication is reported to be suitable method to produce SLN of 60–380 nm size ranges [53].

### 3.7. Optimization of Formulation Variables

For the optimization of the formulation variables, %drug with respect to lipid (drug : lipid) and concentration of emulsifier were selected as independent variables, each at three levels. Particle size, PDI, and drug entrapment were selected as dependent variables (response). The effect of these independent variables on dependable variables was studied using 3^2^ factorial design and Design Expert 9.0.3.1 software (Stat-Ease, Inc., USA). A total of 11 experiments were designed by the software with 2 centre points. Experiments were run in random order to increase the predictability of the model. Full factorial design used for optimization of formulation variables is shown in Table 6.

ANOVA was also applied to determine the significance and the magnitude of the effects of the formulation variables and their interactions. ANOVA for Response 1 (particle size), Response 2 (PDI), and Response 3 (%entrapment) is shown in Table 7. As observed from Table 7, the model *F* values of 73.31 for Response 1 (PS), 20.94 for Response 2 (PDI), and 34.30 for Response 3 (EE) imply that the models are significant. Linear models were obtained for particle size and PDI, while quadratic model showed the best fit for entrapment efficiency. There is only a 0.01% chance that a “model *F* value” for Response 1 and 0.07% for Response 2 and 3 could occur due to noise. *p* values less than 0.0500 indicate that model terms are significant.

Polynomial equation for particle size in terms of coded factors is

(7) $$\begin{array}{c} PS=+186.35-28.62*A-48.65*B. \end{array}$$

Final equation for PDI in terms of coded factors is

(8) $$\begin{array}{c} PDI=+0.21+0.016*A-0.044*B. \end{array}$$

Polynomial equation for EE in terms of coded factors is

(9) EE=+60.84+10.32∗A−5.40∗B+4.65∗AB−1.36∗A2−3.71∗B2.

**Figure 4:**
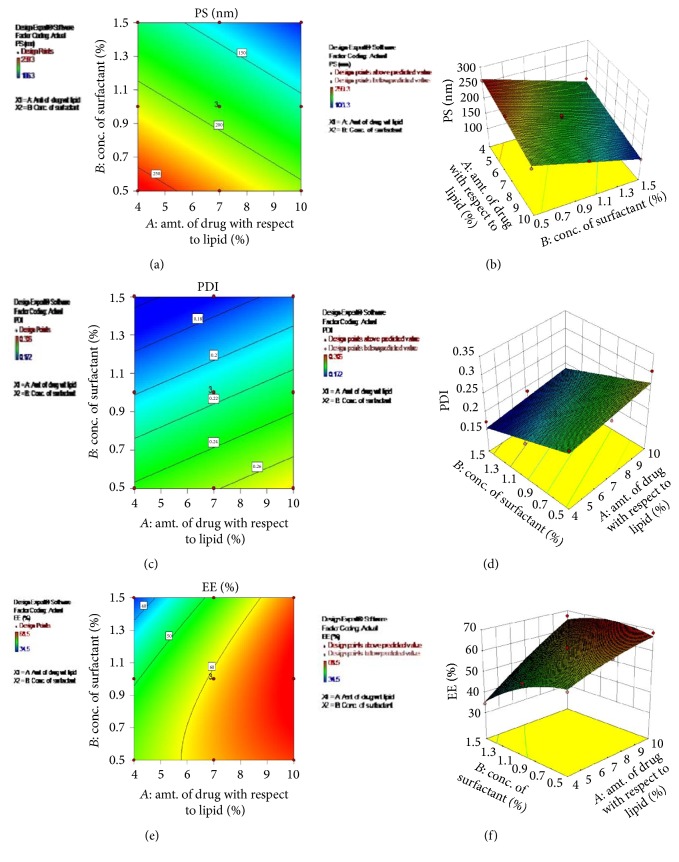
Contour plots and 3D surface plots for formulation variables. (a) Contour plot for particle size. (b) 3D surface plot of particle size. (c) Contour plot for PDI. (d) 3D surface plot of PDI. (e) Contour plot entrapment efficiency. (f) 3D surface plot of entrapment efficiency.

The contour plots and 3D surface plots for particle size, PDI, and %entrapment are shown in Figure 4. It can be observed that particle size was found to reduce with increase in drug : lipid ratio and increase of concentration of surfactant. The results were found in agreement with the reported observations [50]. The reason may be that with increased drug : lipid ratio (decreased lipid content), less surface area of the lipid with high surface tension would have been exposed which could be easily reduced with less surfactant amount. The excess surfactant may be utilized for further reducing the surface tension due to creation of increased surface area due to reduction of particle size. PDI was also observed to reduce with increase in surfactant concentration. This may be because high concentrations of the surfactant would have reduced the surface tension and facilitated the particle partition during homogenization. The increase of the surface area during HPH occurs very rapidly due to reduction in size. The process of a primary coverage of the new surfaces competes with the agglomeration of uncovered lipid surfaces [50, 54].

#### 3.7.1. Experimental Validation of Design Space (for Formulation Variables)

**Figure 5:**
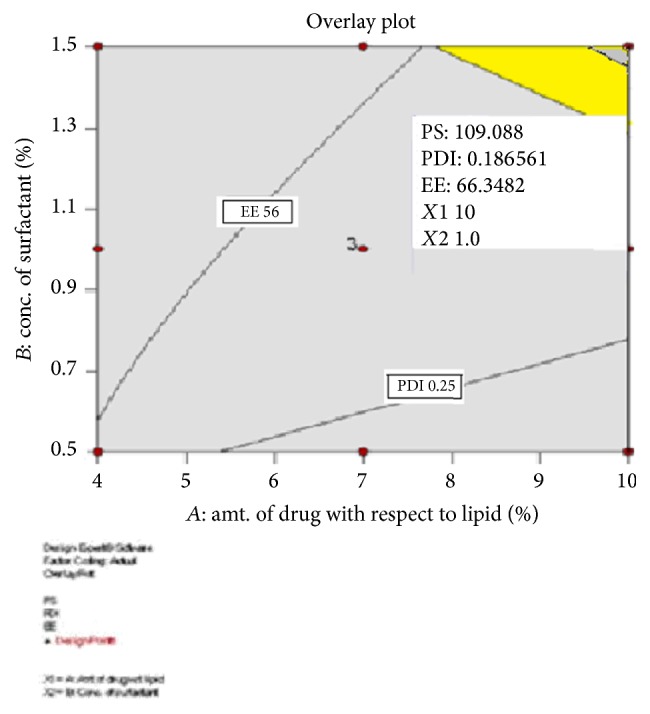
Overlay plot for optimization of formulation variables.

Experimental validation of DoE trials for formulation variables was undertaken by formulation and characterization of nanoparticles at the checkpoint batch suggested by the software.  Figure 5 shows the overlay plot displaying the design space and optimized parameters as checkpoint suggested by DoE software to obtain the desired responses. The observed values (particle size 108.3 nm, PDI 0.172, and EE 64.9%) were comparable with the predicted values (particle size 109.088 nm, PDI 0.186561, and EE 65.3482) establishing the reliability of the optimization procedure.

### 3.8. Evaluation of Optimized Formulation

#### 3.8.1. Particle Size, Polydispersity Index (PDI), Zeta Potential, and Entrapment Efficiency

**Figure 6:**
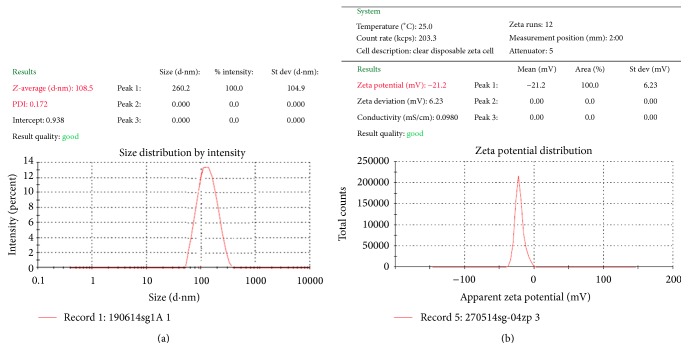
Size distribution and zeta potential distribution of optimized efavirenz nanoparticles.

The average particle size, PDI, zeta potential, and entrapment efficiency of the solid lipid nanoparticles, determined using Malvern Zetasizer Nanoseries Nano-ZS, were found to be 108.3 nm, 0.172, −21.2 mV, and 64.9%, respectively, as shown in Figure 6. Most SLN dispersions produced by high pressure homogenization (HPH) are characterized by an average particle size below 500 nm and low microparticle content. The attainable particle size is especially dependent on the composition and concentration of lipid and emulsifier [50]. As per reported observations, optimum size (224.8–266.3 nm) was achieved for Clozapine loaded SLN with different lipids including tripalmitin and 1% poloxamer concentration [48] and 241 nm for dihydroartemisinin loaded solid lipid nanoparticles when formulated by single-emulsion solvent evaporation technique [30]. Particles of 200 nm were obtained with polyhydroxy surfactants [50], nanoparticles in the range of 180–190 nm were achieved with cetyl palmitate as lipid and are reported to be suitable for targeting to brain [55, 56], and size of up to 140 nm was achieved for cetyl palmitate SLN when the formulation of lipid nanoparticles was optimized with the aid of a computer generated experimental design [49]. In general, *Z*-average size of 20–500 nm is reported depending on the drug, lipid, surfactants, and the formulation technique used [48, 57].

Average particle size of less than 110 nm indicated the suitability of the formulation for administration through various routes with the potential of increased permeability and thus enhanced bioavailability of the poorly soluble drug efavirenz. Low PDI value (<0.2) indicated the narrow distribution of size (monodispersity) and stability of the formulation was indicated by the zeta potential value (−21.2 mV).

Zeta potential is an important physicochemical parameter that influences the stability of nanosuspensions. Extremely positive or negative zeta potential values cause larger repulsive forces, whereas repulsion between particles with similar electric charge prevents aggregation of the particles and thus ensures easy redispersion. In the case of a combined electrostatic and steric stabilization achieved by large molecule weight stabilizers, a minimum zeta potential of ±20 mV is desirable [26].

#### 3.8.2. Transmission Electron Microscopy

**Figure 7:**
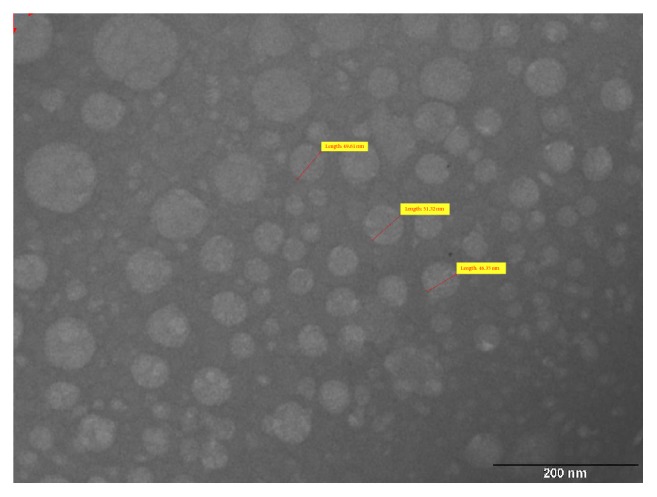
Transmission Electron Microscopic image of efavirenz nanoparticles obtained using transmission electron microscope with CCD camera (TEM Philips Tecnai 20, Holland).

The surface morphology of the optimized SLN was investigated using transmission electron microscope. The image taken using transmission electron microscope with CCD camera (TEM Philips Tecnai 20, Holland) is shown in Figure 7. Spherical particles were observed with drug incorporated in the lipid matrix.

#### 3.8.3. Histopathological Studies

**Figure 8:**
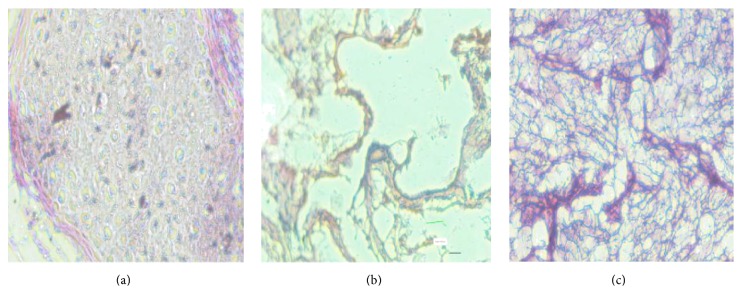
Histopathological conditions of nasal mucosa after treatment with (a) phosphate buffer saline (PBS) pH 6.4. (b) Isopropyl alcohol. (c) SLN formulation.

Histopathological conditions of nasal mucosa after treatment with PBS pH 6.4 (negative control), isopropyl alcohol (positive control), and developed formulation are shown in Figure 8.

No significant damage/harmful effects on the microscopic structure of the nasal mucosa treated with SLN formulation was observed in comparison to that of sample treated with isopropyl alcohol indicating the safety of the SLN formulation for nasal administration. This was in accordance with the results obtained by Seju et al. [34]. Mucosa treated with isopropyl alcohol showed heavy loss of epithelial cells.

#### 3.8.4. Drug Release Profile

**Figure 9:**
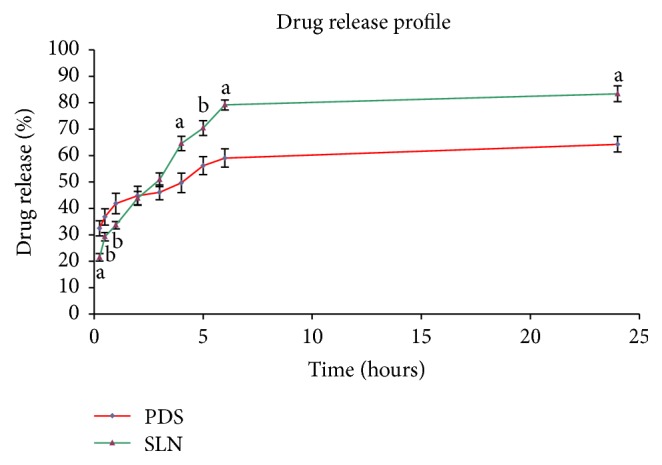
Drug release profile (different letters indicate statistically significant difference relative to PDS; a indicates *p* < 0.01 and b indicates *p* < 0.05).

*In-vitro* drug diffusion profile of the SLN dispersion was obtained by dialysis-bag/dialysis-sac method and was compared with that of plain drug suspension (PDS). The results obtained are as shown in Figure 9. The release of drug from the SLN dispersion was found to be more consistent in comparison to the release from plain drug suspension. It showed initial burst release followed by a prolonged release in accordance with other investigations [35]. 83.4% drug release was observed in 24 hours with SLN dispersion and 79.2% release achieved in 6 hours in comparison to the drug release of 59.1% in 6 hours and total of 64.3% in 24 hours with plain drug suspension. The small size of nanoparticles and the presence of surfactant in the developed formulation may have improved the permeability, wetting, solubilization, and the dissolution of the soluble surfactants to form pores in the matrix and may have played the role for consistent and enhanced release of the drug. Similar results are also observed and reported that nanosized drug delivery systems can enhance nose-to-brain delivery of drugs compared to equivalent drug solutions formulations [33, 37, 58]. Protection of the drug from degradation and/or efflux back into the nasal cavity may partly be the reason for this effect of nanoparticles [9].

#### 3.8.5. *In-Vivo* Studies

The concentration of the drug in plasma and brain was determined after intranasal administration of the developed solid lipid nanoparticulate formulation (equivalent to 0.06 mg efavirenz) and was compared with the concentration of the drug achieved in plasma and brain with the oral administration of the marketed formulation (25 mg powder from capsule dispersed in 1 mL water). The ratio of drug concentration in brain to plasma of 15.61% was achieved with the developed formulation in comparison to 0.104% observed with the oral standard indicating the 150 times more brain targeting efficiency of the formulation through intranasal route which may be because of direct nose-to-brain delivery achieved through integrated olfactory and trigeminal route. The drastic reduction in brain to plasma drug concentration with oral route may be due to first-pass effect, drug degradation in GIT, and presence of BBB. The bioavailable fraction of the drug was calculated to be 0.2454 with the developed formulation while it was found to be 0.0035 with the standard. Relative bioavailability was determined to be 70.11 with the developed formulation indicating 70 times better absorption potential of the efavirenz loaded SLN dispersion in comparison to the orally administered drug powder. This may be attributable to systemic absorption of some amount of drug when administered intranasally. This may be higher in comparison to the orally administered drug due to avoidance of first-pass metabolism, degradation of drug in GIT, and so forth. The results were found to be in accordance with the similar investigations with different drugs for brain targeting [58].

#### 3.8.6. Stability Studies

The stability of the formulation was assessed under different storage conditions as per ICH guidelines and the results obtained are as shown in Table 8.

The developed formulations were found to be stable for 6 months at 5 ± 3°C and 25 ± 2°C/60 ± 5% RH. No change in the physical appearance of the formulation was observed during the stability studies. No significant change in the *Z*-average size, PDI, and zeta potential were observed during the stability studies when analyzed using Student's *t*-test. Thus it can be concluded that efavirenz SLN were stable at long-term stability conditions (5 ± 3°C) as well as accelerated conditions (25 ± 2°C/60 ± 5% RH) for 6 months.

## 4. Conclusion

With the present investigations, it may be concluded that solid lipid nanoparticles of a poorly soluble drug efavirenz were successfully formulated and optimized using the systematic approach of design of experiments (DoE) by high pressure homogenization technique. The intranasal administration of the formulation showed 150 times more brain targeting efficiency and 70 times better absorption potential of the efavirenz loaded SLN dispersion in comparison to the orally administered marketed formulation (capsule). Thus, it may be concluded that the developed formulation has better potential for reducing the plasma viral levels with low dose of efavirenz giving less toxicity as well targeting brain where the HIV virus is reported to harbor. Hence, the developed formulation has the potential for an attempt to completely eradicate HIV reservoir and cure AIDS after the investigations of clinical trials.

## Figures and Tables

**Table 1:** Selection of surfactant on the basis of particle size, PDI and Entrapment Efficiency {^*∗*^Data expressed as mean ± SD (*n* = 3) # indicates statistical significance (*p* < 0.05), PDI: Polydispersity Index}.

S. number	Lipid	Surfactant (1% w/w)	Particle size^*∗*^ (nm)	PDI^*∗*^	Entrapment efficiency^*∗*^ (%)
1	*Tripalmitin*	*Poloxamer 188*	*566.4 ± 7.4* ^*#*^	*0.494 ± 0.2*	*23.93 ± 0.31*
		*(Pluronic F68)*			
2	Tripalmitin	Poloxamer 407	891.1 ± 8.1	0.363 ± 0.3	16.50 ± 0.51
		(Pluronic F127)			
3	Tripalmitin	Poloxamer 245	628.3 ± 9.3	0.488 ± 0.3	18.31 ± 0.57
		(Pluronic P 85)			
4	Tripalmitin	Polysorbate 20	697.4 ± 9.7	0.511 ± 0.4	17.33 ± 0.49
5	Tripalmitin	Polysorbate 60	620.5 ± 8.9	0.500 ± 0.3	20.14 ± 0.63
6	Tripalmitin	Polysorbate 80	601.3 ± 8.5	0.499 ± 0.3	21.23 ± 0.90

**Table 2:** Major peaks observed in the IR spectrum of efavirenz recorded using FT-IR spectrophotometer (Bruker Alpha-One, Bruker Optik, Germany).

Observed (cm^−1^)	Reported (cm^−1^)	Inference
3319.64	3500–3100	N-H stretching
2249.39	2250–2100	C≡C (alkyne)
1750.06	1750–1730	C=O of ester
1602.59	1680–1630	C=O of amide
1498.33	1350–1000	C-N
1036.16	1300–1000	C-O

**Table 3:** Comparison of formulation techniques on the basis of particle size, PDI, and entrapment efficiency {^*∗*^data are expressed as mean ± SD (*n* = 3); # indicates statistical significance (*p* < 0.01). PDI: polydispersity index}.

S. number	Batch number	Technique	Particle size^*∗*^ (nm)	PDI^*∗*^	Entrapment efficiency^*∗*^ (%)
1	SFT01	Solvent evaporation	479.7 ± 13.4	0.373 ± 0.113	42.24 ± 2.3
2	SFT02	*High pressure* *homogenization*	*376.3* ± *9.5*^**#**^	*0.380 *± *0.101*	*64.76* ± *1.9*^***#***^

**Table 4:** Full factorial design with coded and actual values used for optimization of process variables. (independent variable: pressure and number of cycles; dependent variable: particle size and polydispersity index—PDI).

S. number	Batch number	Coded values		Actual values		Particle size (nm)	PDI
		Pressure (bar)	Number of cycles	Pressure (bar)	Number of cycles		
1	OPC01	−1	−1	500	3	501.0	0.469
2	OPC02	0	−1	700	3	403.1	0.440
3	OPC03	1	−1	900	3	309.8	0.412
4	OPC04	−1	0	500	5	457.7	0.386
5	OPC05	0	0	700	5	376.3	0.380
6	OPC06	1	0	900	5	246.1	0.259
7	OPC07	−1	1	500	7	379.9	0.326
8	OPC08	0	1	700	7	352.7	0.359
9	OPC09	1	1	900	7	235.6	0.261
10	OPC10	0	0	700	5	390.2	0.253
11	OPC11	0	0	700	5	405.5	0.301
12	OPC12	0	0	700	5	389.9	0.286
13	OPC13	0	0	700	5	399.3	0.298

**(a):** 

Response 1—PS (particle size) ANOVA for Response Surface Quadratic Model Analysis of Variance table (partial sum of squares—Type III)						
Source	Sum of squares	df	Mean square	*F* value	*p* value Prob > *F*	
Model	64116.53	5	12823.31	42.26	<0.0001	Significant
*A*—pressure	49886.40	1	49886.40	164.42	<0.0001	Significant
*B*—number of cycles	10061.42	1	10061.42	33.16	0.0007	Significant
*AB*	549.90	1	549.90	1.81	0.2202	Not significant
*A* ^2^	2681.72	1	2681.72	8.84	0.0207	Significant
*B* ^2^	73.55	1	73.55	0.24	0.6375	Not significant
Residual	2123.88	7	303.41	—	—	—
Lack of fit	1634.49	3	544.83	4.45	0.0915	Not significant
Pure error	489.39	4	122.35	—	—	—
Cor total	66240.41	12	—	—	—	—

**(b):** 

Response 2—PDI (polydispersity index) ANOVA for Response Surface Quadratic Model Analysis of Variance table (partial sum of squares—Type III)						
Source	Sum of squares	df	Mean square	*F* value	*p* value Prob > *F*	
Model	0.049	5	9.838*E* − 003	5.11	0.0272	Significant
*A*—pressure	0.010	1	0.010	5.37	0.0536	Not significant
*B*—number of cycles	0.023	1	0.023	12.18	0.0101	Significant
*AB*	1.600*E* − 005	1	1.600*E* − 005	8.314*E* − 003	0.9299	Not significant
*A* ^2^	9.659*E* − 005	1	9.659*E* − 005	0.050	0.8291	Not significant
*B* ^2^	0.014	1	0.014	7.25	0.0310	Significant
Residual	0.013	7	1.924*E* − 003	—	—	—
Lack of fit	4.726*E* − 003	3	1.575*E* − 003	0.72	0.5899	Not significant
Pure error	8.745*E* − 003	4	2.186*E* − 003	—	—	—
Cor total	0.063	12	—	—	—	—

**Table 6:** Full factorial design with coded and actual values used for optimization of formulation variables (independent variable: amount of drug with respect to lipid and concentration of surfactant; dependent variable: particle size, polydispersity index (PDI), and entrapment efficiency (EE)).

S. number	Batch number	Coded values		Actual values		Particle size (nm)	PDI	EE (%)
		Amt. of drug with respect to lipid (%)	Conc. of surfactant (%)	Amt. of drug with respect to lipid (%)	Conc. of surfactant (%)			
1	ODS01	−1	−1	4	0.5	259.3	0.250	54.4
2	ODS02	0	−1	7	0.5	246.1	0.247	62.0
3	ODS03	1	−1	10	0.5	194.9	0.306	68.5
4	ODS04	−1	0	4	1.0	175.8	0.172	51.2
5	ODS05	0	0	7	1.0	126.3	0.187	60.5
6	ODS06	1	0	10	1.0	106.3	0.18	66.3
7	ODS07	−1	1	4	1.5	197.4	0.187	34.5
8	ODS08	0	1	7	1.5	191.8	0.214	50.8
9	ODS09	1	1	10	1.5	159.6	0.218	67.2
10	ODS10	0	0	7	1.0	124.5	0.204	62.0
11	ODS11	0	0	7	1.0	137.9	0.197	61.5

**(a):** 

Response 1—PS (particle size) ANOVA for Response Surface Linear Model Analysis of Variance table (partial sum of squares—Type III)						
Source	Sum of squares	df	Mean square	*F* value	*p* value Prob > *F*	
Model	19114.42	2	9557.21	73.31	<0.0001	Significant
*A*-*A*	4913.48	1	4913.48	37.69	0.0003	Significant
*B*-*B*	14200.94	1	14200.94	108.93	<0.0001	Significant
Residual	1042.95	8	130.37	—	—	—
Lack of fit	1024.26	6	170.71	18.27	0.0528	Not significant
Pure error	18.69	2	9.34	—	—	—
Cor total	20157.37	10	—	—	—	—

**(b):** 

Response 2—PDI (Poly Dispersity Index) ANOVA for Response Surface Linear Model Analysis of Variance table (partial sum of squares—Type III)						
Source	Sum of squares	df	Mean square	*F* value	*p* value Prob > *F*	
Model	0.013	2	6.560*E* − 003	20.94	0.0007	Significant
*A*—amt. of drug	1.504*E* − 003	1	1.504*E* − 003	4.80	0.0598	Not significant
*B*—conc. of surf.	0.012	1	0.012	37.08	0.0003	Significant
Residual	2.506*E* − 003	8	3.133*E* − 004	—	—	—
Lack of fit	2.360*E* − 003	6	3.933*E* − 004	5.39	0.1648	Not significant
Pure error	1.460*E* − 004	2	7.300*E* − 005	—	—	—
Cor total	0.016	10	—	—	—	—

**(c):** 

Response 3—EE (Entrapment Efficiency) ANOVA for Response Surface Quadratic Model Analysis of Variance table (partial sum of squares—Type III)						
Source	Sum of squares	df	Mean square	*F* value	*p* value Prob > *F*	
Model	949.81	5	189.96	34.80	0.0007	Significant
*A*—amt. of drug	638.60	1	638.60	115.32	0.0001	Significant
*B*—conc. of surf.	174.96	1	174.96	31.59	0.0025	Significant
*AB*	86.49	1	86.49	15.62	0.0108	Significant
*A* ^2^	4.65	1	4.65	0.84	0.4014	Significant
*B* ^2^	34.78	1	34.78	6.28	0.0541	Not significant
Residual	27.69	5	5.54	—	—	—
Lack of fit	26.52	3	8.84	15.16	0.0625	Not significant
Pure error	1.17	2	0.58	—	—	—
Cor total	977.50	10	—	—	—	—

**Table 8:** Stability study data for the formulation at initial, after 0.5, 1, 2, 3, and 6 months  {^*∗*^data are expressed as mean ± SD (*n* = 3)}.

Temp (°C)/RH (%)	Time (months)	Appearance of nanoparticulate dispersion	Particle size^*∗*^ (nm)	PDI^*∗*^	Zeta potential^*∗*^ (mV)
5 ± 3	0	Transparent with slight bluish	108.5 ± 2.1	0.172 ± 0.003	−21.2 ± 1.9
	0.5	No significant change	109.2 ± 1.9	0.175 ± 0.007	−21.1 ± 2.0
	1	No significant change	109.9 ± 2.0	0.181 ± 0.009	−20.9 ± 1.5
	2	No significant change	110.3 ± 1.8	0.186 ± 0.009	−20.8 ± 1.2
	3	No significant change	110.5 ± 2.3	0.190 ± 0.011	−19.7 ± 2.0
	6	No significant change	112.3 ± 2.8	0.195 ± 0.015	−18.5 ± 1.8

25 ± 2/60 ± 5	0	Transparent with slight bluish	108.5 ± 2.1	0.172 ± 0.003	−21.2 ± 1.9
	0.5	No significant change	106.5 ± 3.2	0.169 ± 0.004	−20.0 ± 1.8
	1	No significant change	109.5 ± 3.5	0.180 ± 0.008	−19.9 ± 2.0
	2	No significant change	111.4 ± 3.7	0.197 ± 0.009	−18.2 + 2.2
	3	No significant change	112.9 ± 2.9	0.205 ± 0.013	−17.5 ± 1.7
	6	No significant change	118.3 ± 2.7	0.209 ± 0.016	−17.0 ± 1.5
